# Catatonia, Pregnancy, and Electroconvulsive Therapy (ECT)

**DOI:** 10.1155/2023/9601642

**Published:** 2023-07-07

**Authors:** Khushbu Gandhi, KieuHanh Nguyen, Maggie Driscoll, Zahid Islam, Siddhartha Maru

**Affiliations:** Department of Psychiatry, Lehigh Valley Health Network, Bethlehem, PA, USA

## Abstract

**Background:**

Catatonia is a neuropsychiatric syndrome, which typically occurs in the context of another psychiatric or medical condition, with a significant morbidity and mortality risk. Significant medical conditions resulting from catatonia include nutritional deficiencies, skin ulcerations, electrolyte disturbances, aspiration pneumonia, and venous thromboembolism. As a result, prompt treatment is required. Gold standard treatment consists of benzodiazepines, followed by electroconvulsive therapy (ECT) if pharmacotherapy alone is ineffective. With pregnancy and catatonia, there is a high risk of adverse maternal/fetal outcomes, and the risks/benefits of treatment must be carefully considered.

**Case:**

Here, we present a case of a young pregnant woman with schizoaffective disorder whose catatonic state was not successfully resolved with lorazepam, therefore requiring ECT. Patient presented to the emergency department at 20 weeks of pregnancy, displaying symptoms of catatonia and psychosis. She was admitted to the inpatient behavioral health unit, where she was treated with lorazepam for catatonia. Treatment occurred in close collaboration with the obstetrics team. While initially, the patient appeared to have a positive response to lorazepam, she became increasingly catatonic with minimal oral intake, mutism, and urinary retention. As a result, she was transferred to the medical floor, where ECT was initiated due to the ineffectiveness of lorazepam. Her catatonia was successfully resolved with 12 total treatments of ECT; there were no adverse effects to the fetus. Patient delivered her baby at 39 weeks with no complications. She continued to receive inpatient psychiatric care until she was stable for discharge to an extended acute care unit.

**Objectives:**

In this report, we will review relevant literature on catatonia in pregnancy, with focus on treatment with ECT.

**Conclusions:**

Though the literature on these topics is limited and typically presented in case reports format, there appears to be a favorable view toward the use of ECT for pregnant catatonic patients. This case could be considered a vital contribution to the literature, as it provides a successful example of treating catatonia in pregnancy with no known adverse effects to the mother or child.

## 1. Introduction

According to the DSM-5-TR, catatonia is a neuropsychiatric syndrome with significant psychomotor disturbances, ranging from motor immobility to excessive motor activity. In order to meet the criteria for catatonia, an individual must display 3 of 12 distinct features: waxy flexibility, stupor, catalepsy, agitation, mutism, negativism, posturing, stereotypies, mannerisms, grimacing, echolalia, and echopraxia [1]. Severe psychomotor agitation, hyperthermia, and autonomic dysfunction are considered to be malignant features and could be rapidly fatal if not appropriately treated [2]. Patients with immobility, staring, mutism, withdrawal, refusal to eat, rigidity, posturing, waxy flexibility, and echolalia/echopraxia are considered to have retarded catatonia, which could lead to medical complications, including dehydration, malnutrition, pulmonary embolism, and deep vein thrombosis [2]. Both excited and retarded catatonic syndromes could lead to malignant features. The pathophysiology of catatonia is poorly understood but is hypothesized to include abnormalities in glutamate and gamma-aminobutyric acid signaling [2].

Given the severity of untreated catatonia, identifying this condition is of utmost importance. Unfortunately, the most overt symptoms, such as posturing, are not always present in patients with catatonia [3]. Currently, there are six different scales available to help identify catatonia: the Rogers Catatonia Scale, the Bush-Francis Catatonia Rating Scale, the Northoff Catatonia Rating Scale, the Braunig Catatonia Rating Scale, the Kanner Scale, and the Modified Rogers Scale [3]. Across the scales, there is a lack of uniformity, difference in applicability, and ease of administration [3]. In 2011, Sienaert et al. [3] published a systematic review of catatonia rating scales; of the six scales, the Bush-Francis Catatonia Rating Scale is the most preferred due to its validity and reliability. This scale includes 23 items, including stereotypy, rigidity, withdrawal, and combativeness, with ratings from 0 to 3 (absent to severe) [4]. The presence of 2 of the first 14 “screening” items indicates a patient has catatonia [5].

According to the DSM-5-TR, catatonia occurs in the setting of another psychiatric condition, such as schizophrenia, bipolar disorder, or major depressive disorder, or in the context of another medical condition, such as neoplasms, encephalitis, and hepatic encephalopathy. Recently, there has been a growing interest in the link between catatonia and autism spectrum disorder (ASD). A significant portion of adolescents and young adults with ASD also have catatonia. The prevalence of catatonia was higher in adolescents and young adults with ASD, who were more passive in social interactions and had impaired expressive language compared to their counterparts [6]. There are also common symptoms that can occur in both ASD and catatonia, including mutism, negativism, stereotypic movements, repetitive behaviors, excitement, and echolalia [7]. This overlap can make it difficult to differentiate between catatonia versus ASD. According to Vaquerizo-Serrano et al. [8], roughly 10% of individuals with ASD had features of catatonia. The catatonia associated with ASD typically presents in late adolescence and occurs more frequently in males. The differentiating feature is that catatonic symptoms are typically new-onset or are secondary to worsening existing symptoms. On the other hand, symptoms of ASD start in the early preschool years. Another consideration is that obsessive-compulsive symptoms can co-occur with ASD and can present as catatonic symptoms with obsessive slowness and counting rituals [8].

Once catatonia is identified, prompt treatment is required, given the high morbidity and mortality risk. Treatment includes benzodiazepines, followed by electroconvulsive therapy (ECT) if the response to benzodiazepines is insufficient [9]. Catatonia becomes further complicated by pregnancy. The use of psychotropic medications during pregnancy is associated with neurodevelopmental effects to the fetus [9]. Pharmacotherapy used during the first trimester often increases the risk of congenital malformations, and those used during the last trimester can increase the risk of drug toxicity or withdrawal effects [9]. In regards to benzodiazepine use during pregnancy, diazepam and chlordiazepoxide are considered safer for use during pregnancy, though not recommended, while alprazolam is not considered a safe option. Available literature recommends that benzodiazepines should be used as monotherapy at the lowest effective dosage for the shortest duration possible, and the daily dosage should be divided into two or three doses to avoid high-peak concentrations [10].

ECT is considered a safe alternative to pharmacotherapy during pregnancy as there is less fetal exposure to the transplacental transfer of chemical substances [9]. However, some providers may be hesitant to use ECT due to potential risks for the fetus. Limited literature exists on catatonic pregnant patients and ECT. Among the literature, there appears to be no consensus on the safety of ECT for use in catatonic pregnant patients. According to a comprehensive literature review by Ward et al. [11], ECT has been safely used in pregnancy over the past 50 years. The rates of miscarriages, congenital anomalies, or neurocognitive disturbances in individuals who received ECT are similar to those in the general population. The most common risks to pregnant women who receive ECT are premature contractions and preterm labor, but these events are infrequent and not clearly linked to ECT [11].

Here, we present a case of a 25-year-old pregnant female, with schizoaffective disorder, who presented with catatonia and was successfully and safely treated with ECT. This case specifically incorporates the treatment of catatonia during pregnancy with ECT. Additionally, it highlights the risks and benefits of benzodiazepines and ECT as the treatment of catatonic symptoms.

## 2. Patient Information

A 25-year-old female with a history of schizoaffective disorder, bipolar type, at 20 weeks of pregnancy, presented with psychosis and catatonia in the setting of medication nonadherence. She was at her obstetrics (OB)/gynecology appointment when the provider noticed her bizarre behavior and agitation. They recommended that her father take her to the emergency department (ED) for an evaluation. In the ED, the patient was disorganized and endorsed hallucinations, both auditory and visual. She was agitated and resistant to medical care. Her father petitioned for involuntary inpatient psychiatric admission, given the severity of her psychosis.

Patient had a history of multiple inpatient psychiatric hospitalizations since childhood, including a suicide attempt via overdose approximately 2 years prior to presentation. Her last inpatient psychiatric hospitalization was approximately 1 year prior to presentation, where she was stabilized on paliperidone palmitate and lithium. At discharge, the patient moved outside of the United States with her husband and discontinued her psychotropic medications. Patient returned to the United States 1 month prior to admission to give birth in the United States. Her condition acutely worsened during the week leading to admission.

## 3. Diagnostic Assessment

The patient was diagnosed with catatonia associated with another mental disorder, schizoaffective disorder, after meeting the following criteria; agitation, mutism, and negativism [1]. Per DSM-5-TR, this diagnosis can be made if a patient meets at least three of the criteria noted in the introduction during the course of a psychotic, bipolar, depressive, or neurodevelopmental disorder [1]. Our patient appeared to be experiencing psychotic symptoms prior to arrival to the ED. Prior to attributing the patient's catatonia to her underlying psychiatric diagnosis, laboratory testing and physical examination were performed, which failed to identify medical conditions contributing to catatonia.

## 4. Therapeutic Interventions

Patient was admitted to the behavioral health unit, and a second opinion on forced medications was completed. Patient showed clinical improvement after receiving two doses of lorazepam 1 mg IM. The patient's mutism and negativism improved, and she was able to ambulate on the unit. Patient was then treated with lorazepam PO/IM 0.5 mg twice daily for catatonia and haloperidol PO/IM 2.5 mg twice daily for underlying psychosis. Lorazepam PO/IM was later increased to 1 mg twice daily, and haloperidol PO/IM was increased to 5 mg twice daily. OB was consulted and agreed with the treatment plan. While the initial response to lorazepam was positive, the patient became increasingly catatonic with agitation that required seclusion, minimal oral intake, mutism, and urinary retention. Lorazepam PO/IM was increased to 1 mg three times daily with no clinical improvement. Electrolyte abnormalities and elevated creatinine kinase were observed. Straight catheterization yielded no urine output.

The patient was transferred to the medical floor and placed on IV fluids with a nasogastric tube for nutrition. Lorazepam IV was increased to 2 mg four times daily without significant improvement of her catatonia, while haloperidol IM was discontinued due to creatinine kinase elevation. Due to the ineffectiveness of lorazepam in resolving her catatonia, ECT was proposed as the next step in treatment. OB was consulted to evaluate the risks of procedure; they recommended proceeding with ECT. Court-ordered ECT was approved for 12 sessions, and she received 10 sessions. The fetus was monitored via nonstress test prior to and after each ECT session. There were no adverse effects to the fetus over the course of treatment. By the first ECT treatment, lorazepam IV was decreased to 1 mg daily and 0.5 mg nightly, and risperidone liquid 0.5 mg twice daily was started to target her continued psychosis. However, when risperidone liquid was increased to 1 mg twice daily, her liver function tests started to rise, so risperidone was discontinued. Lorazepam IV was changed to 0.5 mg three times daily, and paliperidone PO 1.5 mg nightly was initiated. Within a few days, the patient's liver function tests started to rise again, and paliperidone was discontinued. By the fourth ECT session, there was significant improvement of her catatonia, with increased social interactions and oral intake. After 10 treatments, her catatonia resolved. However, her psychosis became more prominent. Paliperidone PO was reinitiated and titrated slowly.

## 5. Follow-Up and Outcomes

She was transferred back to the inpatient psychiatric unit for further treatment following the completion of 10 treatments. Within 2 weeks, she decompensated again despite further titration of paliperidone PO to 12 mg nightly. She received two additional sessions of ECT with gradual improvement of her condition. Patient was transferred from the inpatient psychiatric unit to the OB delivery unit at 39 weeks and had a normal spontaneous vaginal delivery and uncomplicated postpartum course. The baby was discharged with the patient's sister. The patient was maintained on paliperidone PO 12 mg nightly and transferred back to the inpatient psychiatric unit. Upon readmission to the unit, she continued to struggle with delusions, psychosis, and disorganized thoughts. Lithium PO 300 mg twice daily was added to the regimen for further mood stabilization. Mirtazapine PO 7.5 mg nightly was added to the regimen for sleep. Lorazepam PO 1 mg twice daily was continued as needed for anxiety. Lithium PO was titrated to 750 mg daily, and her lithium level was therapeutic at 0.9. Patient was discharged to an extended acute care unit for structured medication management and additional time for symptom reduction. Her regimen on discharge consisted of paliperidone PO 12 mg nightly, lithium PO 300 mg daily and 450 mg nightly, lorazepam PO 1 mg twice daily as needed for anxiety, and mirtazapine PO 7.5 mg nightly as needed for sleep.

## 6. Discussion

Literature on catatonia in pregnancy and the use of ECT is scarce and typically presented in case report format. These case reports tend to favor ECT and suggest this form of treatment as an effective and safe method to resolve catatonia when pharmacotherapy has failed [9]. Though there has been limited data on catatonia, pregnancy, and ECT, there has been literature on using ECT to treat other serious psychiatric illnesses in pregnancy [12]. According to Ray-Griffith et al. [13], ECT has been the treatment for a variety of psychiatric conditions since 1935, including unipolar depression, bipolar depression, acute suicidal ideation with plan, mania, catatonia, and psychosis. Remission rates have been up to 87% for depression and mania and 61% for primary psychotic disorders [13].

Catatonia requires prompt treatment, given the significant morbidity and mortality risk it poses if left untreated. With pregnancy, immediate treatment of catatonia is required, given the potential harm for both mother and fetus. Medical complications of catatonia during pregnancy include maternal dehydration, muscular atrophy, electrolyte disturbances, nutritional deficiencies, skin ulcerations, aspiration pneumonia, and venous thromboembolism [14]. These medical concerns can have a significant impact on the fetus' ability to survive and thrive. Additionally, the withdrawal and mutism associated with catatonia in pregnancy can leave the patient unable to communicate signs of maternal or fetal distress and/or labor, as well as impair mother–infant bonding after delivery [14].

Initial treatment of catatonia involves high-dose benzodiazepines, up to 20 mg of lorazepam per day. Resolution of catatonia occurs in up to 80% of cases [15]. ECT is the second-line treatment of catatonia if the benzodiazepine monotherapy is ineffective [14]. In general, ECT is considered the gold standard treatment of severe mental illness in pregnant patients to avoid the potential teratogenicity of perinatal exposure to psychotropic medications.

There is evidence that benzodiazepine use during pregnancy is associated with an increased risk of oral cleft, and exposure during the third trimester is associated with neonatal abstinence syndrome, hypotonia, apnea, temperature dysregulation, and poor suckling [14]. However, while ECT is considered the safer option for pregnant patients, the procedure itself has been associated with rare occurrences of placental abruption, hematuria, vaginal bleeding, preterm labor, fetal heart rate (FHR) decelerations, and miscarriages [14]. The most common maternal adverse event from ECT is premature labor [12, 16]. Other maternal adverse events include vaginal bleeding and abortion when ECT is done in the first trimester [12]. Patients with placenta previa, chronic abruption, or subchorionic hematoma should be closely monitored due to the increased risk of vaginal bleeding [13]. Uterine contractions, cesarean sections, and premature labor can occur when ECT is done in the second and third trimesters [12]. The rates of serious adverse events, such as stillbirth, neonatal death, and fetal malformation, appear higher than that of the general population [12]. As a result of these potential adverse events, it is important to closely monitor the mother and fetus during and after ECT treatments, including the use of ultrasonography between treatments, cardiotocography to monitor for fetal cardiac malformations, pre- and post-ECT tocolytic treatment to prevent preterm labor in the second trimester, tilt position for mother to reduce the risk of gastric reflux in the third trimester as well as inhalation anesthesia to reduce uterine contraction [12].

There are also limited studies that monitored fetal vitals during the ECT treatments. In the systematic review of case studies by Leiknes et al. [12], some studies had no fetal monitoring, while others had ultrasonography, FHR, and/or Doppler monitoring between treatment sessions. The most common fetal adverse event during ECT is FHR reduction which occurred in 43% of the participants [12]. However, it is unclear whether the FHR reduction was secondary to the ECT or the anesthetic agent [16]. The American College of Obstetrics and Gynecology recommended that prior to 24 weeks of gestation, auscultation of FHR rate should be done before and after each ECT session [17]. After 24 weeks of gestation, intraoperative fetal monitoring can be done. If prolonged bradycardia is seen on FHR monitoring, then resuscitative measures such as oxygen supplementation, intravenous hydration, and left lateral decubitus position should be done [17].

In our report, we present a case of catatonia in pregnancy that was successfully treated when lorazepam was ineffective. While the patient initially responded to 2 mg of lorazepam, her catatonia worsened, and she was unresponsive to doses as high as 8 mg daily. Given the severity of her catatonia, with minimal oral intake, mutism, and urinary retention, ECT was proposed as the next step in treatment due to the risk of continued catatonia for the patient and fetus. Patient received 12 total sessions of ECT, with the resolution of her catatonia symptoms. As treatment occurred, her fetus was monitored by nonstress test prior to and after each ECT session; each nonstress test was negative for adverse effects. Our patient vaginally delivered her child at 39 weeks with a noncomplicated postpartum course. Her child was healthy on delivery with no known adverse effects from the ECT treatment. Unfortunately, we were not able to obtain information regarding the infant's health postdelivery and therefore cannot determine if there were any long-term effects from ECT.

Though our discussion highlights the possible maternal and fetal adverse events from undergoing ECT, our report exemplifies a successful case of catatonia in pregnancy with no known detrimental effects to the mother or child. In doing so, we add to the scarce literature of catatonia in pregnancy and propose that ECT can be an efficacious and safe treatment for both mother and child. Additionally, it is important to highlight that close collaboration with OB, both prior to and during the ECT process, would be beneficial for minimizing the risk to the mother and fetus.

## 7. Conclusion

Catatonia in pregnancy poses a significant risk to both mother and fetus, given the associated medical complications. Gold standard treatment continues to be an administration of benzodiazepines, followed by ECT if pharmacology therapy alone is unable to resolve the catatonia. Given the scarcity of literature on catatonia in pregnancy, there remains limited data on the use of ECT to treat this condition; however, greater literature exists regarding the use of ECT in pregnancy for other serious mental illnesses, such as severe depression and mania. Our review of the existing literature led us to the conclusion that ECT can be used in the pregnant population, once the risks and benefits have been carefully evaluated, due to the concern of adverse maternal/fetal outcomes. In our report, we present a case of a pregnant female with catatonia whose symptoms progressed to reduced oral intake, mutism, and urinary retention, despite the use of benzodiazepines. Given the high morbidity and mortality risk for both the patient and her child, the decision was made to pursue ECT. With 12 total treatments, her catatonic symptoms resolved with no known side effects to the patient or her fetus. With our case, we provide evidence that ECT can be used for catatonic pregnant patients, with minimal to no adverse outcomes for both the mother and fetus. As a result, we believe our case can be a vital contribution to the limited case reports and studies available at this time. Overall, we recommend the use of ECT in catatonic pregnant patients, especially when symptoms require rapid resolution, with careful collaboration with all providers involved.
